# Methodology for rigorous modeling of protein conformational changes by Rosetta using DEER distance restraints

**DOI:** 10.1371/journal.pcbi.1009107

**Published:** 2021-06-16

**Authors:** Diego del Alamo, Kevin L. Jagessar, Jens Meiler, Hassane S. Mchaourab

**Affiliations:** 1 Department of Chemistry and Center for Structural Biology, Vanderbilt University, Nashville, Tennessee, United States of America; 2 Department of Molecular Physiology and Biophysics, Vanderbilt University, Nashville, Tennessee, United States of America; 3 Institute for Drug Discovery, Leipzig University Medical School, Leipzig, Germany; Bogazici University, TURKEY

## Abstract

We describe an approach for integrating distance restraints from Double Electron-Electron Resonance (DEER) spectroscopy into Rosetta with the purpose of modeling alternative protein conformations from an initial experimental structure. Fundamental to this approach is a multilateration algorithm that harnesses sets of interconnected spin label pairs to identify optimal rotamer ensembles at each residue that fit the DEER decay in the time domain. Benchmarked relative to data analysis packages, the algorithm yields comparable distance distributions with the advantage that fitting the DEER decay and rotamer ensemble optimization are coupled. We demonstrate this approach by modeling the protonation-dependent transition of the multidrug transporter PfMATE to an inward facing conformation with a deviation to the experimental structure of less than 2Å C_α_ RMSD. By decreasing spin label rotamer entropy, this approach engenders more accurate Rosetta models that are also more closely clustered, thus setting the stage for more robust modeling of protein conformational changes.

## Author summary

Proteins transition between different conformations during function. Double Electron-Electron Resonance (DEER) spectroscopy enables the direct observation of structural rearrangements that underpin these transitions. Typically, histograms of distances between spin labels, called distance distributions, are measured under different conditions. Structural rearrangements that underlie conformational transitions are manifested by changes in the averages and widths of the distance distributions. To transform these distance distributions into restraints for modeling alternate protein conformations, we developed an algorithm in the modeling suite Rosetta for direct analysis of DEER primary data that yield the optimum ensemble of spin label positions in space, referred to as rotamers, that account for the data. We benchmarked the effectiveness of this algorithm using experimental data collected in two proteins, the model system T4 Lysozyme and the multidrug transporter PfMATE in an outward-facing conformation. We then used optimized spin label rotamers to model the inward-facing conformation of PfMATE from the starting outward-facing conformation. Our results demonstrate substantial improvements in both precision and accuracy among the resulting models. Further improvement of this strategy will enable modeling of protein conformational changes involving complex modes of movements.

This is a *PLOS Computational Biology* Methods paper.

## Introduction

Distance measurements between pairs of spin labels by Double Electron-Electron Resonance (DEER) spectroscopy have been utilized extensively to investigate the structures and dynamics of proteins[1–4] and the assembly of protein-protein complexes[5–8]. At the fundamental level, DEER measures magnetic dipolar coupling to infer the distributions of distances between two or more spin labels[9,10]. A two-step process typically interprets these distances as spatial restraints describing the protein backbone structure. First, the echo-decay time traces are transformed into distributions consisting of distance components characterized by a mean and width[11–15]. Second, these distributions are compared to those predicted using one of several strategies, ranging from generic rotamer libraries[16–18], explicitly modeled pseudoatoms[1,19,20], or explicitly modeled spin label side chains[21–27]. However, these strategies tend to overestimate the dynamics of flexible probes such as the commonly used methanethiosulfonate spin label (MTSSL). Therefore, the predicted distributions are broad relative to the experimental ones[18,20,28–31], which hinders DEER-based evaluation of protein structures or complexes as well as mapping of protein conformational changes. The latter can be obscured entirely if modeled distribution widths exceed distance changes observed between spin labels[1]. Another layer of complications in modeling of conformational changes arises if the ensemble of spin label rotamers is allowed to reconfigure, hence providing a low energy pathway to account for changes in distance distributions that originate from backbone movements. Collectively, these caveats limit the accuracy and precision of molecular models generated from DEER restraints.

Several algorithms have recently been developed to refine ensembles of spin label rotamers by employing multilateration[16,32–36]. Multilateration refers to the determination of an object’s position in three-dimensional space given its distance from a constellation of points; common applications include the positioning of electronic devices using the Global Positioning System and of earthquake epicenters using time-of-arrival data[37]. To utilize this approach to position spin label rotamers requires both a high-resolution starting structure and a set of DEER distance data consistent with that structure. However, a unique challenge in this endeavor is that spin labels are flexible relative to the protein backbone. As a result, the ensembles characterizing their positions must be refined simultaneously for all spin labels in a given protein model.

Molecular dynamics simulations have been used to determine a set of optimized rotamers from explicitly modeled spin labels restrained by experimental distance distributions[14,21,38,39]. Alternatively, rotamer libraries have been precomputed and reweighed using either Monte Carlo[32,35], singular value decomposition[34], or nonlinear least-squares minimization[33]. The positions of these labels can, in turn, be used to more precisely locate paramagnetic ligands or metal ions[35,36,40,41], as well as make small-scale refinements to protein structures[16,42]. To our knowledge, however, none of these methods have demonstrated that these optimized rotamers can lead to improvements in modeling conformational changes.

Furthermore, these methods generally do not address unique factors confounding multilateration of spin labels. First, the width of a distribution reflects disorder in the solid state as a result of backbone and spin label side chain dynamics at room temperature. Existing multilateration methods generally ignore the former, by assuming the distribution is explained entirely by spin label dynamics[16,35], or both, by extracting the peak distance from the distribution and discarding the width[42]. Second, relying on distance distributions rather than time domain data propagates assumptions intrinsic to the method used for the transformation of the latter[10,14]. Depending on the noise level of the experimental measurements, this step can distort true components or introduce ghost components to the distribution. Finally, although DEER distributions are often reported with confidence bands to reflect the uncertainty inherent to this transformation[14,15,43], they are generally taken at face value when used for rotamer multilateration. This incorrectly implies that experimental uncertainty is uniformly distributed across the dataset and can lead to rotamers that over- or underfit the DEER distributions. Collectively, these obstacles prevent the positioning of spin label ensembles in three-dimensional space and complicate the confidence with which such ensembles can be used for subsequent modeling purposes.

To address these issues, we developed and implemented, as part of the RosettaDEER module[20], an algorithm that combines rotamer multilateration[33,35,41] for pairs sharing common spin labeling sites with direct analysis of DEER time traces. The algorithm calculates a weighted distribution of “pseudo-rotamers”, or inflexible coarse-grained side chains, capable of recapitulating large experimental datasets collected using DEER. Importantly, this algorithm goes beyond comparable methods by refining these ensembles using raw data in the time domain, rather than distance distributions calculated *a priori*, thus avoiding the loss of information that can occur as result of data transformation. Using experimental data collected in the model system T4 Lysozyme and the multidrug transporter PfMATE, we demonstrate that this algorithm is able to fit time domain data as effectively as widely-used DEER data analysis programs. Integrated with Rosetta, these rotamers ensembles yield substantial improvements in both accuracy and precision of modeling the outward-to-inward isomerization of the multidrug transporter PfMATE, thus reinforcing the notion that coupling analysis of primary data with rotamer optimization is a superior approach for restrained modeling of protein conformational states.

## Results and discussion

### Overview of the multilateration algorithm

The algorithm capitalizes on the concept of pseudo-rotamers, which are simplified representations of the spin label designed to maximize computational efficiency[20]. A pseudo-rotamer models the spin label side chain as a centroid atom representing the nitroxide ring and its unpaired electron, yielding predicted distance distributions that are comparable to full-atom depictions. Unlike explicit depictions of the spin label used in all-atom simulations, ensembles of pseudo-rotamers do not interact with one another; as a result, the dynamics of spin labels close in space are fully independent. However, in principle, any rotamer library can be used for the multilateration strategy described here[17,18,22,26,27,30].

The transformation of DEER data to distance distributions is an ill-posed mathematical problem necessitating the use of either regularization[15,44,45], parametric modeling[13–15], neural networks[46], or other methods[11,43,47,48]. Because these methods have intrinsic approximations which could interfere with rotamer ensemble determination, we elected to fit the raw experimental data directly using an iterative simulated annealing strategy that 1) measures all pairwise distances between pseudo-rotamers, 2) converts each distance distribution into a DEER decay, and 3) calculates the intermolecular dipolar coupling contribution by nonlinear least-squares minimization. Different levels of noise between DEER traces linked by multilateration were normalized using estimates obtained from each signal’s corresponding imaginary component[21]. The algorithm prioritized the generation of parsimonious ensembles by minimizing the total number of pseudo-rotamers with nonzero weights using the Akaike Information Criterion-corrected (AICc)[49,50]. This metric, which allows for regularization in rotamer space rather than the distance domain, was guided by the heuristic that the flash-freezing process sharpens the distribution of rotamers that contribute to the DEER signal[51,52]. Finally, to account for backbone heterogeneity and the expectation of smoothness in the distance domain, simulated distributions were broadened by a magnitude corresponding to the residues’ intrinsic flexibility, as reported by their respective crystallographic B-factor values[53,54].

### Data analysis benchmark

**Fig 1:**
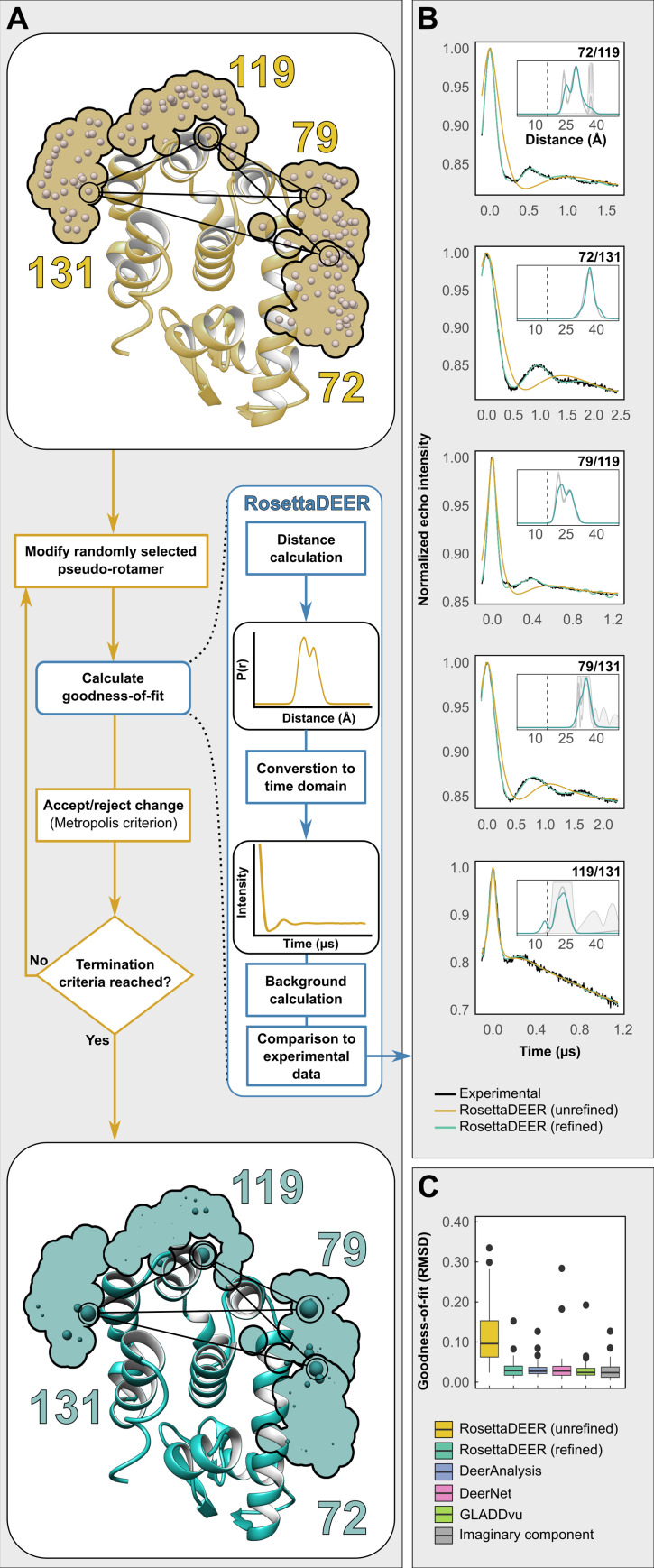
**A)** Distribution of pseudo-rotamers, shown as spheres, at four representative residues in T4 Lysozyme prior to (top, gold) and following (bottom, teal) refinement by multilateration. A flow chart detailing the iterative steps of pseudo-rotamer refinement using RosettaDEER is shown between the two T4L structures. **B)** Five representative DEER traces in T4 Lysozyme used for multilateration, alongside simulated DEER traces prior to (yellow) and following (teal) refinement. Insets: Simulated DEER distributions following pseudo-rotamer refinement alongside reference distributions with 95% confidence bands (calculated using GLADDvu and shown in grey). **C)** Goodness-of-fit evaluated from the RMSD between simulated and experimental DEER traces comparing RosettaDEER to other analysis programs.

We benchmarked this method using experimental DEER data collected in two model proteins, T4 Lysozyme[31,55] (PDB: 2LZM) and the MATE multidrug transporter PfMATE[56–58] in its outward-facing conformation (PDB: 6GWH). The extracellular and intracellular spin label pairs of PfMATE were treated independently since they did not share residues in common. These three DEER datasets consisted of 65 restraints between 47 residues; a subset of the restraints in T4 Lysozyme is shown in Fig 1A. We note that unlike the benchmarks used in other multilateration methods, these restraints were highly interconnected; half of the residues were spin labeled in three or more DEER pairs, and in the most extreme case, two residues in T4 Lysozyme were spin labeled across seven pairs (S1 Fig). For each of the three datasets, the RosettaDEER multilateration algorithm was executed for 1000 replicas, with each replica yielding refined pseudo-rotamer ensembles at every spin labeled site.

We compared the resulting fits to those obtained using GLADDvu[14], DeerAnalysis[44], and DeerNet[46], which are programs that analyze DEER data using Gaussian mixture models, Tikhonov regularization, and feed-forward neural networks, respectively. Although other analysis methods are available, we believe these represent a sufficiently diverse range of analytical approaches for the purposes of comparison. We found that the optimum rotamer ensembles, selected by the AICc, could recapitulate the experimental DEER traces as effectively as each of these programs (Figs 1B, 1C and S2–S5 and S1–S3 Appendices). The mean squared errors obtained by the best fit were not statistically different from those obtained by any of these three methods, or from the noise estimated from the imaginary component (Student’s paired one-tailed t-test with Bonferroni correction). However, unlike the latter methods, the interconnectedness of the spin label pairs allowed our algorithm to couple pseudo-rotamer parametrization to the analysis of DEER data in the time domain.

### Distance distribution benchmark

**Fig 2:**
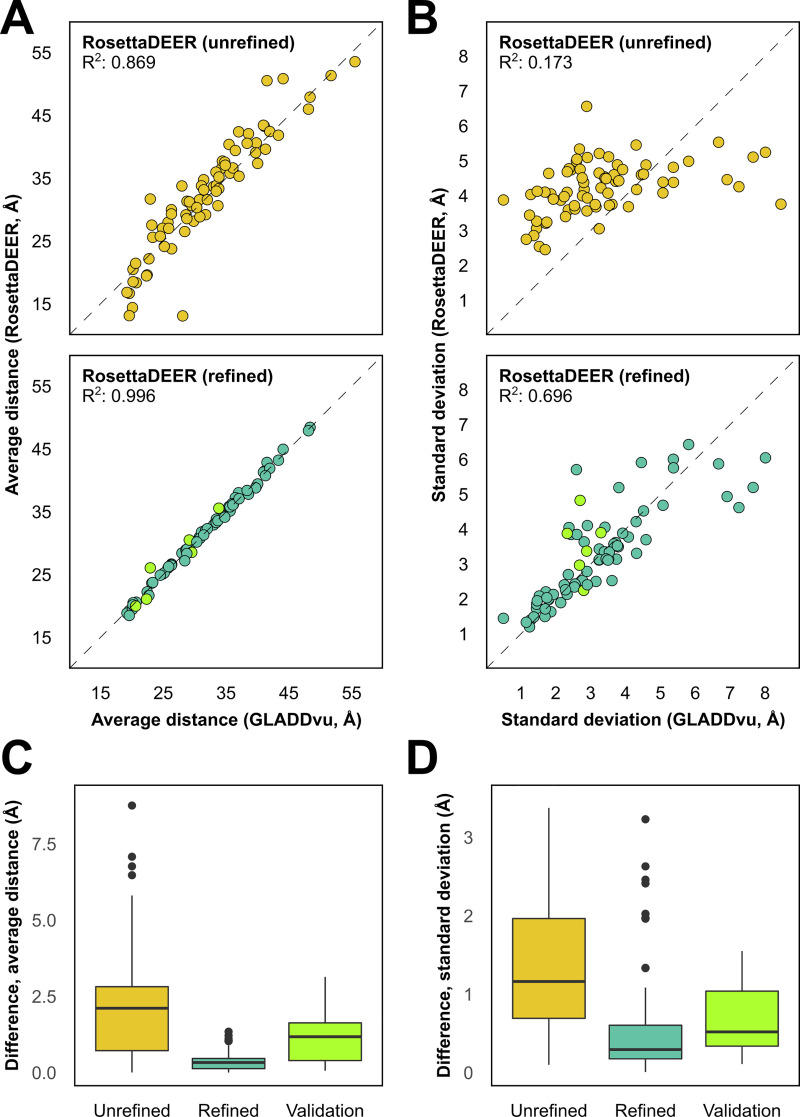
Evaluation of average distances **(A)** and distribution widths **(B)** between pseudo-rotamers prior to (top) and following (bottom) refinement by multilateration. T4 lysozyme distributions omitted from multilateration are shown in light green. **C and D)** Boxplots showing the difference between values obtained using GLADDvu and values simulated between pseudo-rotamer ensembles prior to and following refinement.

We anticipated that the analysis of DEER data by multilateration would yield distance distributions similar to those obtained using traditional methods. Consistent with this expectation, distributions between refined pseudo-rotamers in both T4L and PfMATE showed remarkable agreement with those obtained using the three methods mentioned above (see Figs 1B insets for examples and S3–S5 Figs for all distributions). For example, the average values of these distributions were within 0.5 Å of those obtained using GLADDvu for 60 of the 65 restraints (Fig 2A). Additionally, the widths of 52 of these restraints were within 0.5 Å of those obtained using GLADDvu. Discrepancies occurred for broad distributions or long distances (because the information content in the time domain is not as well-defined) or components less than 15 Å (because these distances minimally contribute to the DEER signal). Additionally, we uncovered differences when comparing the widths of these distributions to those obtained using DeerAnalysis, likely resulting from small “ghost” side peaks frequently observed in regularization. Discrepancies were also observed when comparing these distributions to those obtained using DeerNet, which yielded widths clustered between 2.5 and 4.5 Å (S6 Fig).

Finally, the uncertainty of these distributions was calculated from the five pseudo-rotamer ensembles with the lowest AICc values. The resulting confidence bands, which capture 95% of the variation in the distance distributions, are qualitatively comparable to those obtained using GLADDvu, DeerAnalysis, and DeerNet (S7 Fig).

To further validate the algorithm, we simulated distance distributions for six T4L spin label pairs which were excluded from the multilateration dataset. We observed that the median error between the average distance values fell by 50% (Fig 2; full distributions shown in S8 Fig) using the refined rotamers. By contrast, the standard deviations did not significantly sharpen, and their values are similar to those observed prior to refinement. Notably, the uncertainty of these distributions is greater than those of the distributions included in the training set.

### Modeling of PfMATE’s conformational changes using refined pseudo-rotamers

**Fig 3:**
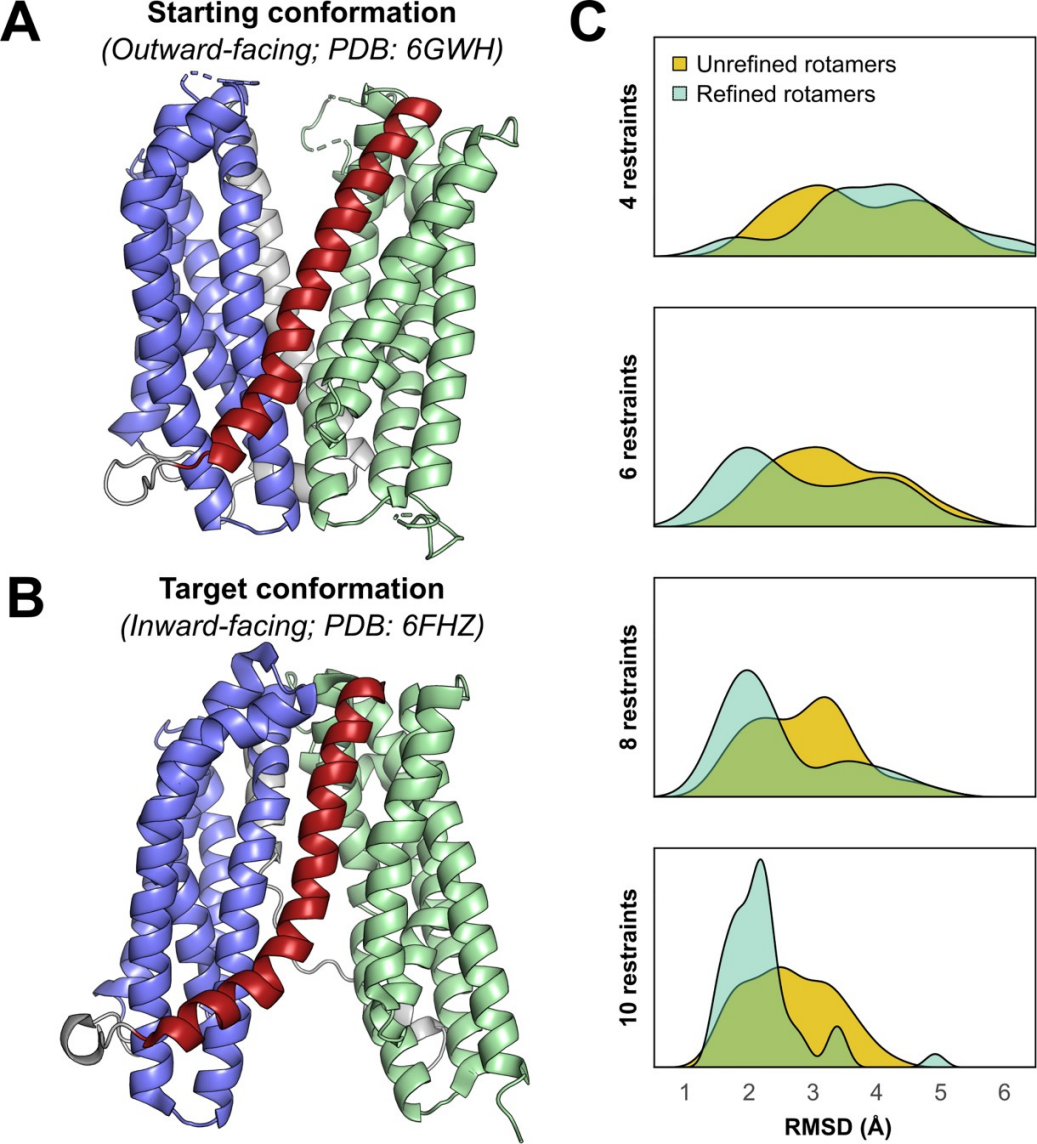
Modeling the outward-to-inward conformational change in the multidrug transporter PfMATE. **(A)** Outward-facing and **(B)** inward-facing crystal structures of PfMATE. N- and C-terminal domains are shown in purple and green, respectively, and TM7 is shown in red. **(C)** RMSD values of the ten best-scoring models for each of four sets of restraints relative to the inward-facing conformation using either pseudo-rotamers refined by multilateration (teal) or unrefined pseudo-rotamers available by default (yellow).

While the results above demonstrate the robustness of the multilateration algorithm in identifying optimal spin label pseudo-rotamer ensembles, the central question is whether these provide superior restraint quality for modeling conformational changes. To address this question, we modeled the isomerization of PfMATE between outward- and inward-facing conformations[56,57] (OF and IF, shown in Figs 3A and 3B, respectively), both of which were determined by x-ray crystallography. The two conformations differ primarily in the relative orientations of the N- and C-terminal domains resulting from changes in the backbone dihedral angles of transmembrane helix 7 (TM7). Of direct relevance to the question addressed here, distance distributions between pairs of spin labels measured at pH 7.5 and pH 4.0 were shown to be consistent with the OF and IF conformations, respectively[58].

We generated several thousand models, using Rosetta[59] without DEER restraints, by perturbing TM7 and found that none of the built-in membrane protein scoring functions[60–63] could identify the inward-facing state by score alone (S9 Fig and S4 Appendix) even if it was included in the initial model set. Thus, from a Monte Carlo modeling perspective, the OF-to-IF conformational transition can be sampled, but not necessarily identified, without experimental data.

To test the notion that DEER restraints interpreted with the refined pseudo-rotamers can drive convergence of Rosetta modeling, we identified spin label pairs where the EPR lineshape showed minimal changes upon a pH shift from 7.5 to 4.0 (see ref. [58] for all data), supporting the approximation that the spin label rotamer ensembles are invariant and thus were not allowed to reconfigure during Rosetta modeling. From these pairs, 40 sets of restraints were generated, each of which consisted of one to ten spin label pairs (S1 Table). Using scoring functions to assess the agreement with the DEER restraints (see *Materials and Methods*), the OF-to-IF conformational transition was modeled by perturbing the dihedral angles of TM7. DEER distributions were simulated using either the pseudo-rotamers ensembles refined by multilateration or the unrefined ensembles available to RosettaDEER by default. Agreement with the experimental distributions was evaluated by the overlap between the experimental and simulated distance distributions. Similarity to the inward-facing crystal structure was quantified by the root mean squared deviation (RMSD) of the alpha carbons excluding TMs 1 and 7.

We observed a striking contrast between the effectiveness of the refined and unrefined ensembles (Fig 3C and S4 Appendix). The default rotamer library did not effectively improve the average RMSD of the ten lowest-scoring models beyond 2.0–3.5 Å. By contrast, the use of multilaterated pseudo-rotamers converged upon inward-facing models with a 1.5–2.5 Å C_α_ RMSD using restraints obtained from the same spin label pairs.

**Fig 4:**
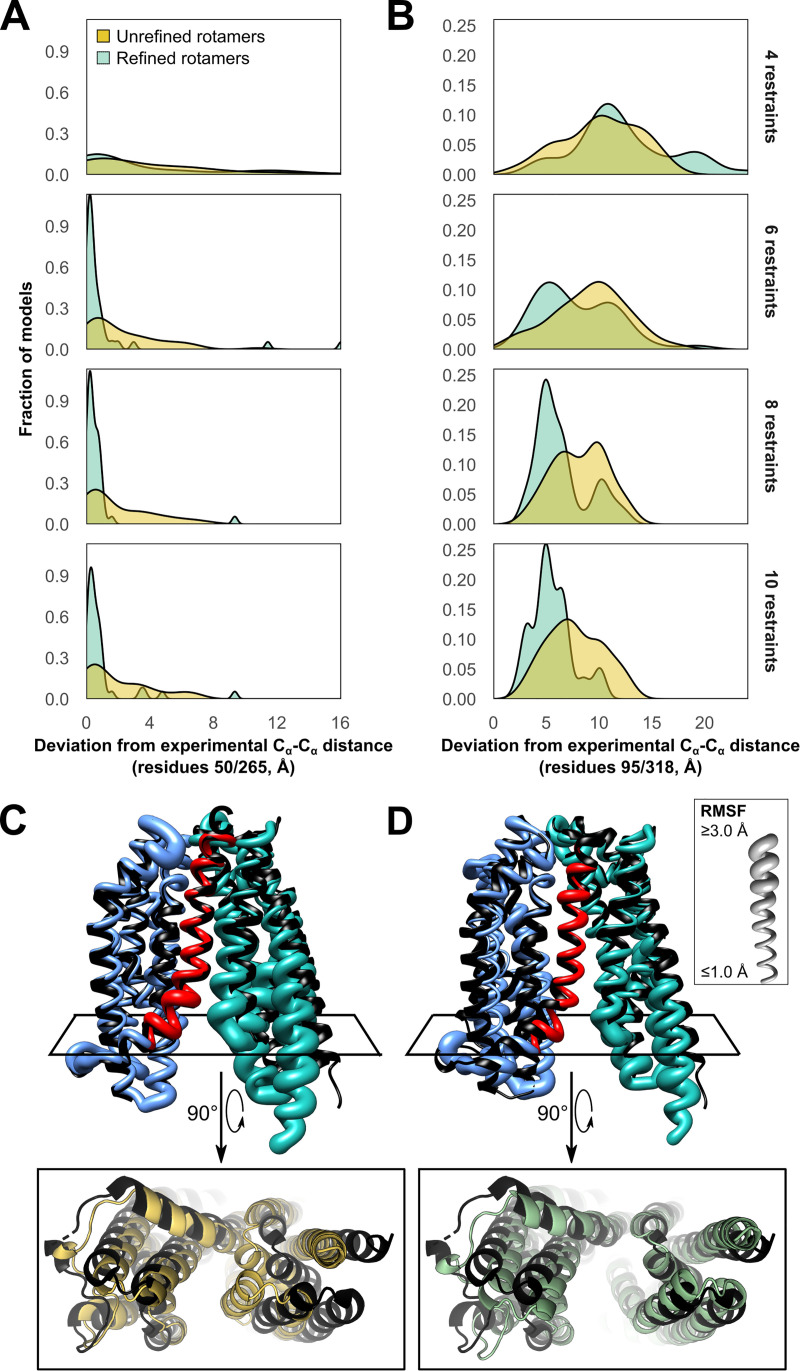
Models of PfMATE obtained using multilaterated rotamers more closely resemble the inward-facing crystal structure than those obtained using default rotamers. Deviation between C_α_-C_α_ distances observed between representative pairs of residues on the **A)** extracellular and **B)** intracellular sides of the crystal structure (PDB: 6FHZ) and the corresponding distances predicted from each of the best-scoring models. **(C and D)** Best-scoring inward-facing models of PfMATE obtained using ten restraints either with pseudo-rotamers available by default (left) or with those refined by multilateration (right). Inward-facing crystal structure shown in black. Ribbon thickness corresponds to the C_α_ root mean squared fluctuation among the top ten models. Bottom: The best-scoring models obtained using default rotamers (left) were less inward-open than those obtained using multilaterated rotamers (right).

Alongside these improvements in accuracy, the sharper range of RMSD values among these models suggested that multilateration improved model precision. Distributions of representative distances across the intracellular and extracellular sides of the top ten models (Figs 4A and 4B) revealed that, when using the default pseudo-rotamers, a majority of these models failed to close the extracellular cavity and were far less inward-open than the crystal structure (Fig 4C), even when ten restraints were used. By contrast, the best-scoring models obtained using refined pseudo-rotamers deviated less drastically from the crystal structure. Nonetheless, these models were virtually all less inward-open than the crystal structure, consistent with shorter-than-expected experimental DEER measurements on the intracellular side at pH 4.0[58] (Fig 4D).

### Concluding remarks

Our results highlight a general strategy to substantially improve the quality of models obtained from EPR restraints. We envision that the main application of this strategy is to model alternate conformational states starting from an experimental structure and a set of interconnected DEER data. By implementing this algorithm in Rosetta, we hope to encourage its use for a wide variety of modeling applications, such as protein-protein docking and *de novo* folding. Moreover, further development of this approach, as well as extensive use of multilateration in the design of spin label pairs, will open the door to modeling proteins where conformational changes are defined by more complex modes of motion.

## Materials and methods

### Overview of the model-based approach

The objective of the RosettaDEER multilateration algorithm is to fit a set of DEER data by weighting the nitroxide pseudo-rotamers available to each spin-labeled residue in a protein structural model. Each replicate of the algorithm independently generates a unique set of pseudo-rotamer ensembles for each spin-labeled residue. For clarity throughout this text, we will refer to these outputs as "coordinate models", to differentiate them from the starting structural models. The space accessible to the unpaired electron of each residue’s spin label is divided into fifty discrete pseudo-rotamers, which are shown as small spheres in Fig 1A. RosettaDEER then identifies and removes pseudo-rotamers that clash with the protein backbone. Each residue’s ensemble of pseudo-rotamers represents a probability density function of the space accessible to the unpaired electron of that residue’s spin label. As a result, following refinement using this algorithm, the weights of a coordinate model’s pseudo-rotamers for any given residue are tightly coupled to those of other residues.

In this study we focus our attention on coordinate models with high parsimony. For example, coordinate models capable of recapitulating DEER traces using only one pseudo-rotamer per residue are prioritized over those with two or more. However, if the DEER trace indicates a broad and multimodal distribution, additional pseudo-rotamers may be necessary to improve the goodness-of-fit. The total number would ideally be no greater than the minimum required to fit the data, and multiple combinations of pseudo-rotamers may be equally consistent with the data. We identified parsimonious coordinate models using the Akaike Information Criterion-corrected (AICc)[49,50,64]:

$$AICc=-2\;\ln(L(\hat{\theta }|D))+2K+\frac{2K(K+1)}{n_{total}-K-1}$$ (1)

This metric balances two competing objectives of 1) fitting the experimental data as well as possible and 2) simplifying the model as much as possible. The leftmost term, goodness-of-fit, is expressed as the maximum likelihood estimate of the coordinate model with parameters **θ** given the experimental DEER data ***D*** and is described below. The middle and rightmost term express the complexity of the model, with the variable *K* corresponding to the total number of parameters in the coordinate model and *n*_*total*_ corresponding to the total number of time points in the experimental DEER data. *K* includes the number of pseudo-rotamers with nonzero weights, as well as the number of parameters required to fit the intramolecular DEER data in the time domain. The rightmost term, which converges to zero as the data-to-parameter ratio increases, serves as further regularization in modeling cases where less experimental data is available (in this case corresponding to the number of time points in all DEER traces). Overall, the AICc quantifies the expectation that few spin label rotamers contribute to the distance distribution.

### Detailed description of the multilateration algorithm

The multilateration algorithm is implemented in Rosetta[59] as part of the RosettaDEER package and can be run using RosettaScripts[65]. It uses an iterative simulated annealing approach and is therefore non-deterministic. As a result, it obtains diverse sets of solutions when executed multiple times. However, there is no guarantee that the global minimum solution is obtained using this algorithm.

The positions of the pseudo-rotamers are kept fixed in space throughout the duration of the algorithm, e.g., they are reweighted, rather than moved. Initial positions are obtained from the nitroxide bond midpoints of each rotamer in the Rosetta MTSSL rotamer library following clash evaluation[22]. At the start of the algorithm, one of these pseudo-rotamers is randomly chosen for each residue and has its weight set to 1; the rest have weights set to zero.

The algorithm then proceeds as follows:

- The weight of a randomly chosen pseudorotamer is modified by a randomly chosen number. Initially this value ranges uniformly from -0.1 to 0.1.
- The weight change is applied, and the resulting sum-of-squared residuals is calculated as discussed below.
- Any move that decreases the sum-of-squared residuals is accepted, while any move that increases it is accepted with the following probability (with *iter* being the current iteration):

$$p_{accept}=\exp\left(-\frac{\ln\left(L(\theta_{iter+1}|D)\right)-\ln\left(L(\theta_{iter}|D)\right)}{k_{B}T}\right)$$ (2)

- The Boltzmann temperature *k*_*B*_*T* starts at 1.5 and asymptotically approaches zero with each iteration as the algorithm proceeds. A total of 2500 trials per round are performed per DEER trace in the dataset. However, each round is aborted if 500 consecutive trials fail to sample an improvement.
- At the end of each round, the temperature *k*_*B*_*T* is raised to 1.5. If no improvements were sampled, the magnitude of the weight changes made to coordinates is reduced by a factor of $\sqrt{10}$. Once this magnitude reaches 10^−4^, the algorithm is concluded.

For PfMATE, we used a non-three-dimensional background model to fit the intermolecular contribution of the experimental signal. This required a modification to the algorithm in which the first round of optimization was performed using a three-dimensional background. The first time *k*_*B*_*T* was reset to 1.5, this restriction was removed. Otherwise, the dimensionality of the intermolecular background coupling was found to immediately drop to a value of 2, trapping the solution in a local minimum.

### Simulation of DEER distance distributions

To simulate distance distributions between two spin-labeled residues *u* and *v*, pairwise distances were measured between all coordinates belonging to each residue. To account for backbone heterogeneity, each of these measurements were then broadened by a value equal to the pairwise root mean square fluctuation (RMSF) as inferred from the crystallographic isotropic B-factor of the residues’ C_α_ atoms:

$${RMSF}_{u}=\sqrt{\frac{3B_{u,C\alpha }}{8\pi^{2}}}$$ (3)

$${RMSF}_{uv}=\sqrt{{RMSF}_{u}^{2}+{RMSF}_{v}^{2}}$$ (4)

The result is equivalent to the convolution of the original distribution with a Gaussian distribution with a width of RMSF_uv_. Regions of proteins with higher B-factors, such as loops, have previously been found to exhibit a greater degree of backbone flexibility in solution[53,66,67]. Failure to account for backbone flexibility could potentially overstate the intrinsic dynamics of the spin label and decrease the precision of the models generated using the pseudo-rotamers obtained this way. We did not normalize the experimental B-factors to account for differences in experimental crystallographic resolution, since such differences may reflect variations in the backbone disorder of different proteins.

### Evaluating coordinate models obtained from raw DEER traces

In all examples discussed in this manuscript, the data ***D*** comprises *N* decay traces (V_exp_), e.g., ***D*** = {*V*_*exp*,*1*_, *V*_*exp*,*2*_,…, *V*_*exp*,*N*_}, with the *i*th decay trace consisting of *n*_*i*_ time points for a total of *n*_*total*_ experimental time points among all experimental traces. In this case, the likelihood of the model was evaluated by the noise-normalized sum-of-squared residuals to the experimental data:

$$\ln\left(L(\theta |D)\right)=-\frac{n_{total}}{2}*\ln\left(\frac{1}{n_{total}}\sum_{i=1}^{N}\sum_{i_{t}=1}^{n_{i}}{\left(\frac{V_{exp,i}(t_{i_{t}})-V_{intra,i}(t_{i_{t}}|\theta )}{\sigma_{i}}\right)}^{2}\right)$$ (5)

Here *σ*_*i*_ is the standard deviation of the noise corresponding to the *i*th decay trace, *V*_*exp*,*i*_(*t*_*i*_) refers to the experimental data at the *i*_*t*_th time point of decay trace *i*, and $V_{intra,i}(t_{i_{t}}|\theta )$ refers to the value of the simulated data in decay trace *i* at time point *i_t_* given the model parameters **θ**. The values of *σ*_*i*_ were calculated from the imaginary component of each DEER trace. Normalizing the data to the noise was necessary to satisfy the assumption that the sum of squared residuals is independently and identically distributed. Forgoing this correction led to overfitting of noisier DEER traces and underfitting of less noisy traces.

Simulation of DEER traces occurred in three steps. First, the distance distributions were obtained from the model coordinates as described above. Second, the intramolecular form factor was calculated for each time point $t_{i_{t}}$:

$$V_{intra,i}(t_{i_{t}}|\theta )=\sum_{j=1}^{m}P_{sim,i}(r_{j}|\theta )\int_{0}^{\frac{\pi }{2}}\sin\left(x\right)*cos(\frac{(1-3{cos}^{2}x)*{\mu_{0}\mu }_{B}^{2}g^{2}t_{i_{t}}}{4\pi \hbar {r_{j}}^{3}})dx$$ (6)

Here, *g* is the electron g-factor, *μ*_*0*_ is the vacuum permeability constant, *μ*_*B*_ is the Bohr magneton, $t_{i_{t}}$ is the *i*_*t*_th time point in microseconds, *r* is the bin distance in nanometers, and *x* is the angle between the bulk magnetic field and the interspin vector.

In the third step, the modulation depth, background slope, and dimensionality (in the case of PfMATE) were determined using nonlinear least-squares minimization. This background was modeled as follows:

$$B(t)=exp(-{\left(kt\right)}^{d/3})$$ (7)

The parameter *d* refers to the dimensionality of background coupling and was constrained to a value of 3.0 for T4 Lysozyme and to between 2.0 and 3.5 for PfMATE. In the latter case, we generally obtained values ranging from 2.0 to 2.5. These parameters were determined using an initial search as previously described and were fine-tuned throughout the duration of the algorithm using the Levenberg-Marquardt algorithm.

### Determination of distance distributions

We used GLADDvu[14] and DeerAnalysis2019b[44] to fit the data and obtain distance distributions. Each DEER trace was truncated by 500 ns to avoid fitting artifacts. Sum-of-Gaussian distributions were obtained with GLADDvu using the interior point method. The distribution with the lowest Bayesian Information Criterion was selected. Distributions were also obtained using Tikhonov regularization with an L-curve criterion with default settings, as well as the generic DeerNet neural network ensemble, using DeerAnalysis2019b[46]. Confidence bands and/or error margins were obtained using the delta method for GLADDvu, the Validation tool for Tikhonov regularization, and built-in ensemble statistics for DeerNet.

### Application to T4 Lysozyme and PfMATE

The algorithm as described above was applied to T4 Lysozyme[55] (PDB: 2LZM) and outward-facing PfMATE structure[56] (PDB: 6GWH). For PfMATE, the data were further separated into the extracellular restraints and the intracellular restraints. The algorithm was executed one thousand times for each of these three datasets. Each of the one thousand coordinate models were scored using the AICc (Eq 1).

### Modeling the OF-to-IF conformational change of PfMATE

Modeling the outward-to-inward conformational change of PfMATE was achieved using a Monte Carlo fragment insertion approach implemented in RosettaScripts. This protocol randomly changes the backbone dihedral angles of certain residues chosen at random to match those of a similar stretch of residues found in protein structures deposited in the PDB. Only residues 1–50 and 241–268 were perturbed. Peptide fragments were obtained from a July 2011 version of the PDB using the Robetta web server[68] with homologous protein structures removed. The fragment insertion protocol was executed 1000 times in RosettaScripts[65] using the *score3* scoring function and was repeated for 5000 cycles. The Boltzmann temperature was set to 1.0. The following scoring function was then used to quantify the similarity between the experimental and simulated DEER distributions:

$$S_{DEER}=-\sum_{i=1}^{N}ln(\sum_{j=1}p_{sim,j}p_{exp,j})$$ (8)

If the event that an experimental and simulated distribution did not overlap, the inner term resolves to ln(*0*). Under these circumstances, this value was automatically set to -87.0, which is equivalent to the natural logarithm of the lowest non-negative value that can be described by a single-precision floating point number.

## Supporting information

S1 Fig Number of DEER restraints per spin-labeled residue across T4 Lysozyme and PfMATE. (TIFF)

S2 Fig All DEER traces determined by multilateration are shown in red. Experimental DEER traces are shown in black. (TIFF)

S3 Fig All DEER distance distributions determined by multilateration are shown in black. DEER distributions calculated using GladdVU are shown in green, with the shaded regions indicating 95% confidence intervals. Distance values shorter than 15 Å (indicated by the dashed line) were not used to simulate DEER traces.
(TIFF)

S4 Fig All DEER distance distributions determined by multilateration are shown in black. DEER distributions calculated using DeerAnalysis are shown in blue, with the shaded regions obtained using the validation tool.
(TIFF)

S5 Fig All DEER distance distributions determined by multilateration are shown in black. DEER distributions calculated using DeerNet are shown in pink, with the shaded regions obtained using ensemble statistics.
(TIFF)

S6 Fig Comparison of average and standard deviation values obtained when fitting DEER data collected in pfMATE and T4 Lysozyme to values obtained using DeerAnalysis and DeerNet. Long-distance fitting artifacts were removed from fits obtained using DeerAnalysis. These fits appeared to overstate the standard deviation values relative to GLADDvu, whereas those obtained using DeerNet appeared to be biased toward certain width values.
(TIFF)

S7 Fig Shaded regions depict 95% confidence intervals, and line represents the mean distribution. Ensembles were selected using the AICc.
(TIFF)

S8 Fig Comparison of DEER distance distributions used to validate pseudo-rotamers obtained using the RosettaDEER multilateration algorithm. Distributions obtained using GLADDvu and RosettaDEER are shown in green and grey, respectively. Confidence bands for RosettaDEER depict the five best sets of pseudo-rotamers.
(TIFF)

S9 Fig Rosetta energy functions for membrane proteins cannot identify the inward-facing conformation of PfMATE. In all three cases, the lowest-energy models are fully occluded from both sides of the membrane. RMSD is measured from the inward-facing crystal structure (PDB: 6FHZ); the first 50 residues were omitted.
(TIFF)

S1 Table List of restraints used for scoring intermediate PfMATE models. (DOCX)

S1 Appendix Summary of experimental DEER measurements collected in T4 Lysozyme and PfMATE. (XLSX)

S2 Appendix Coordinates of top five ensembles of pseudo-rotamers in T4 Lysozyme. (TXT)

S3 Appendix Coordinates of top five ensembles of pseudo-rotamers in PfMATE. (TXT)

S4 Appendix Scores of intermediate PfMATE models. (CSV)

S5 Appendix Supplemental methods. (DOCX)
