# Supplementary figures and images for: Methodology for rigorous modeling of protein conformational changes by Rosetta using DEER distance restraints
Source: PLoS Comput Biol. 2021 Jun 16;17(6):e1009107. doi: 10.1371/journal.pcbi.1009107 (PMC8238229; doi:10.1371/journal.pcbi.1009107)

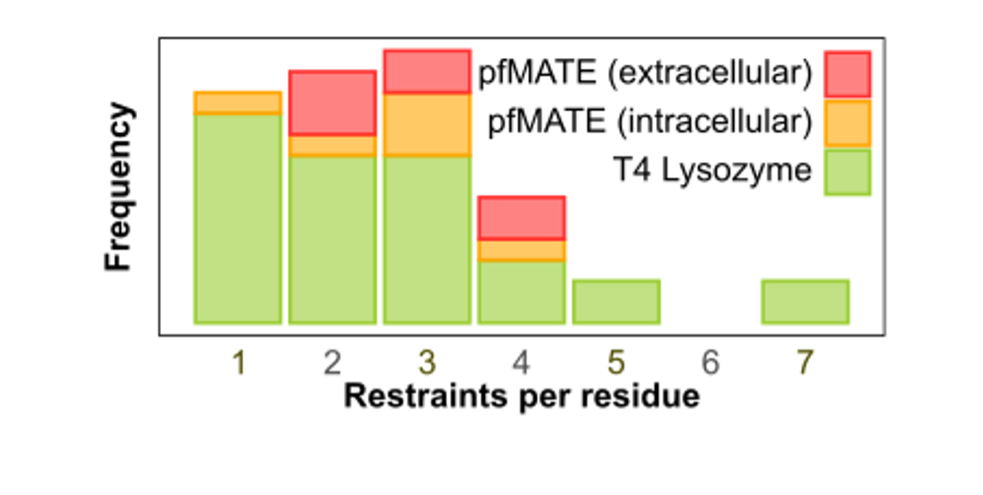

Supplement: S1 Fig — (TIFF) [file pcbi.1009107.s001.tiff]

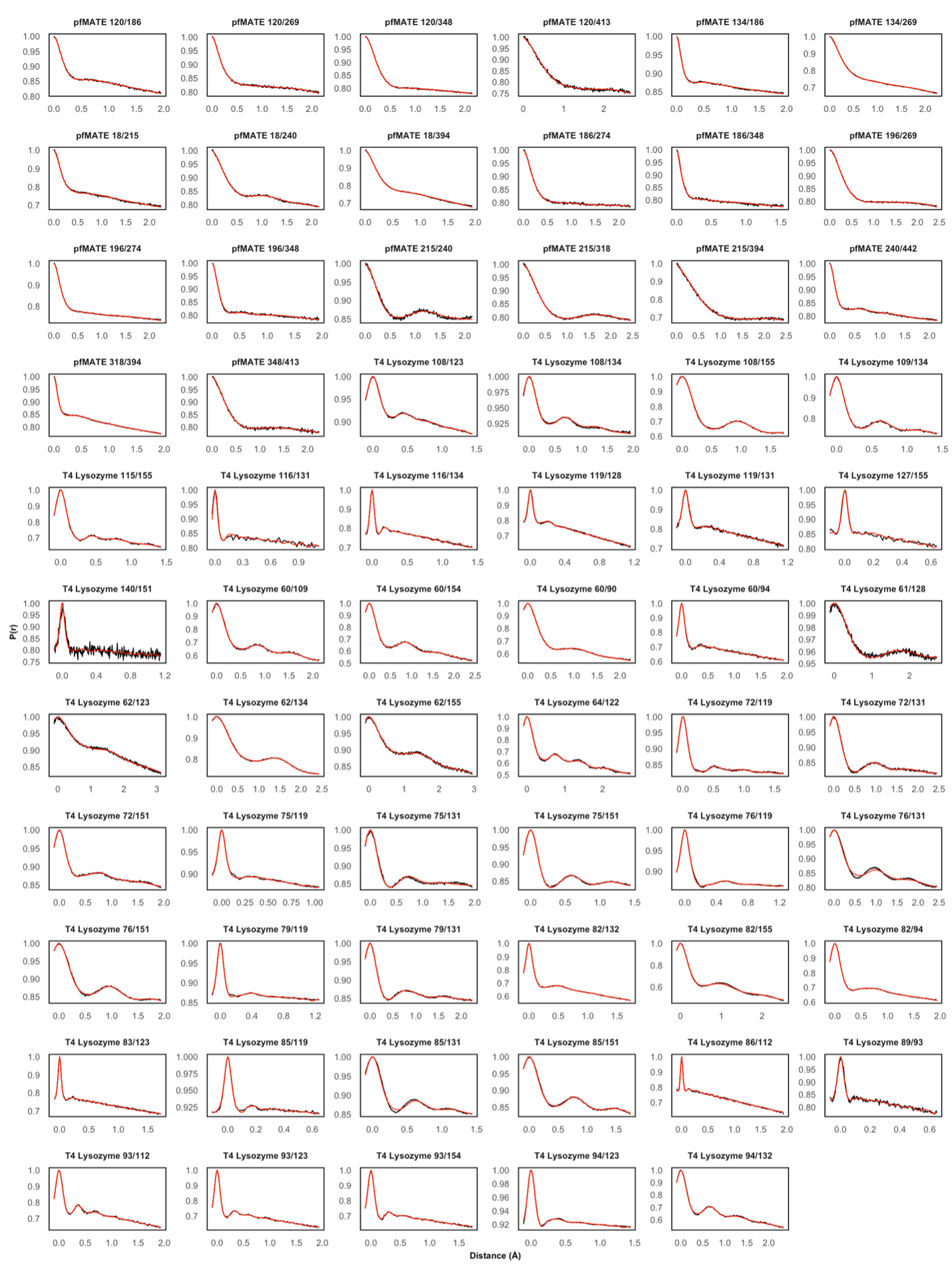

Supplement: S2 Fig — (TIFF) [file pcbi.1009107.s002.tiff]

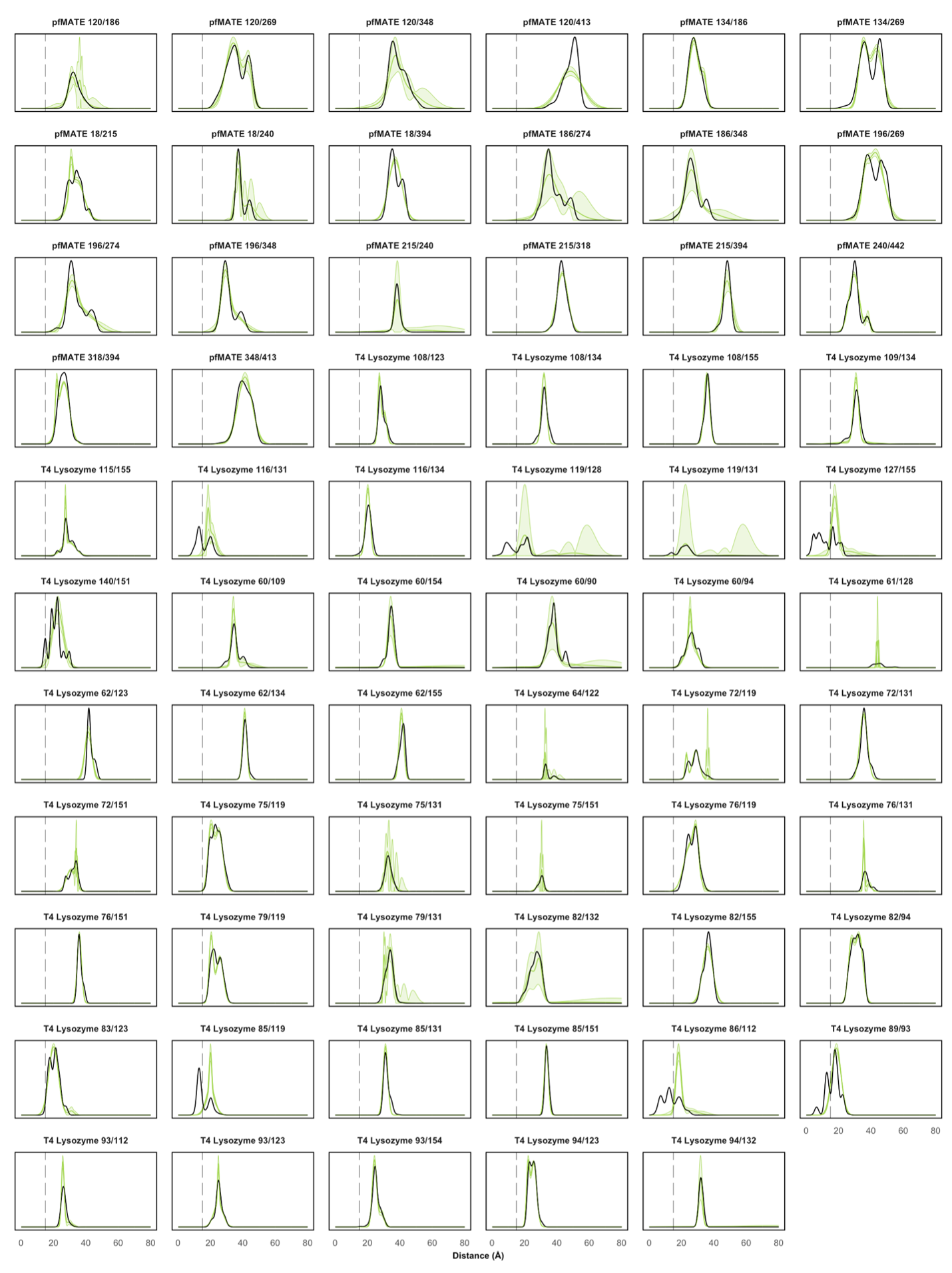

Supplement: S3 Fig — DEER distributions calculated using GladdVU are shown in green, with the shaded regions indicating 95% confidence intervals. Distance values shorter than 15 Å (indicated by the dashed line) were not used to simulate DEER traces. (TIFF) [file pcbi.1009107.s003.tiff]

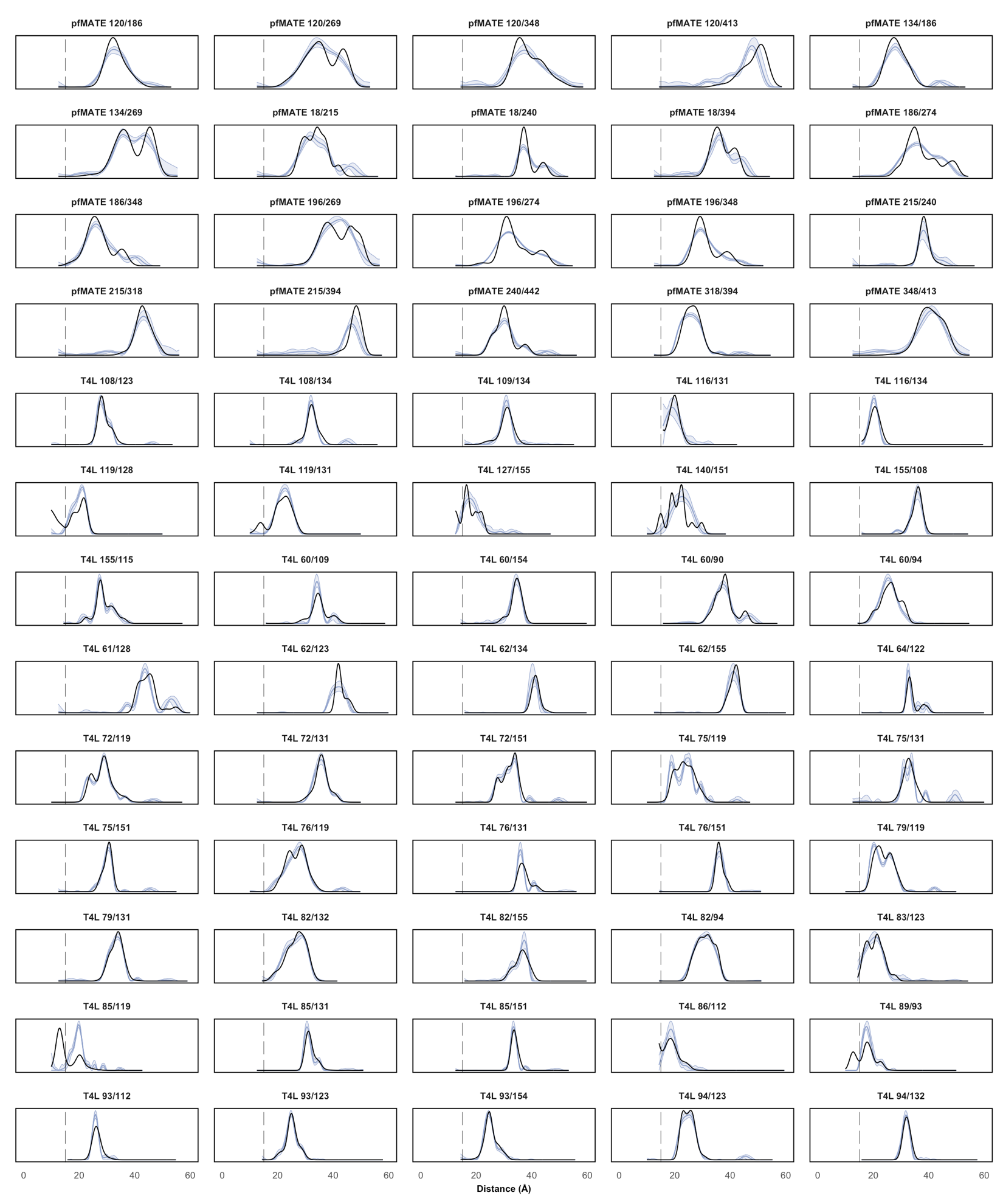

Supplement: S4 Fig — DEER distributions calculated using DeerAnalysis are shown in blue, with the shaded regions obtained using the validation tool. (TIFF) [file pcbi.1009107.s004.tiff]

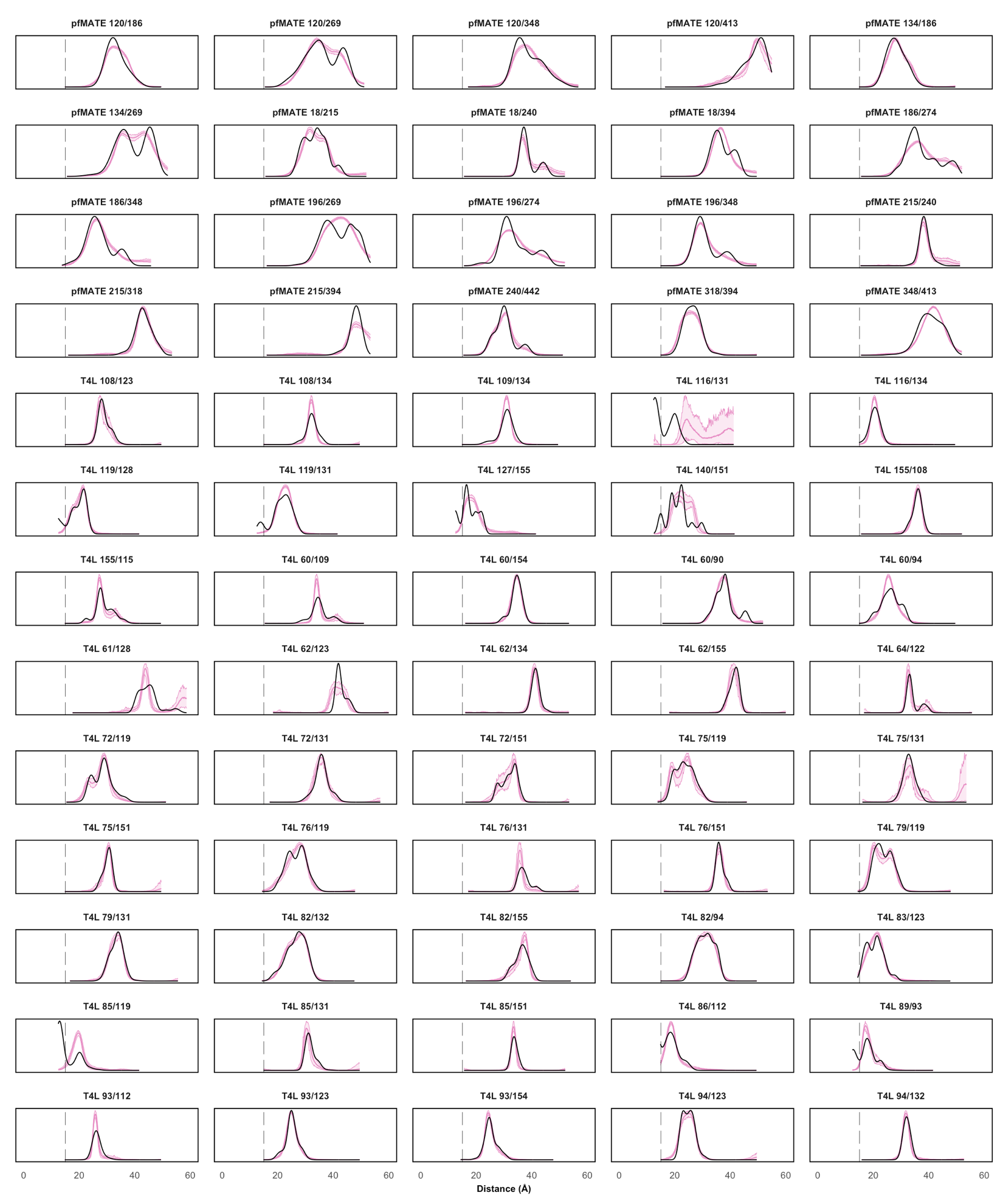

Supplement: S5 Fig — DEER distributions calculated using DeerNet are shown in pink, with the shaded regions obtained using ensemble statistics. (TIFF) [file pcbi.1009107.s005.tiff]

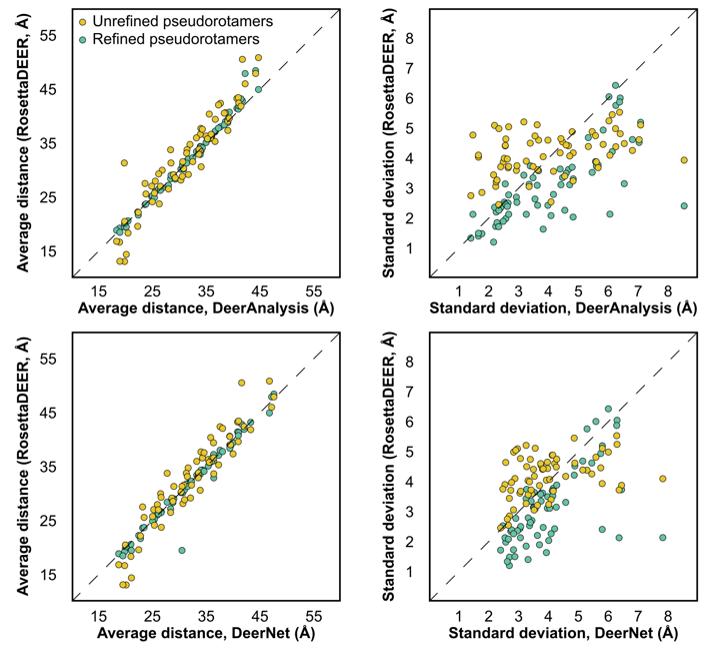

Supplement: S6 Fig — Long-distance fitting artifacts were removed from fits obtained using DeerAnalysis. These fits appeared to overstate the standard deviation values relative to GLADDvu, whereas those obtained using DeerNet appeared to be biased toward certain width values. (TIFF) [file pcbi.1009107.s006.tiff]

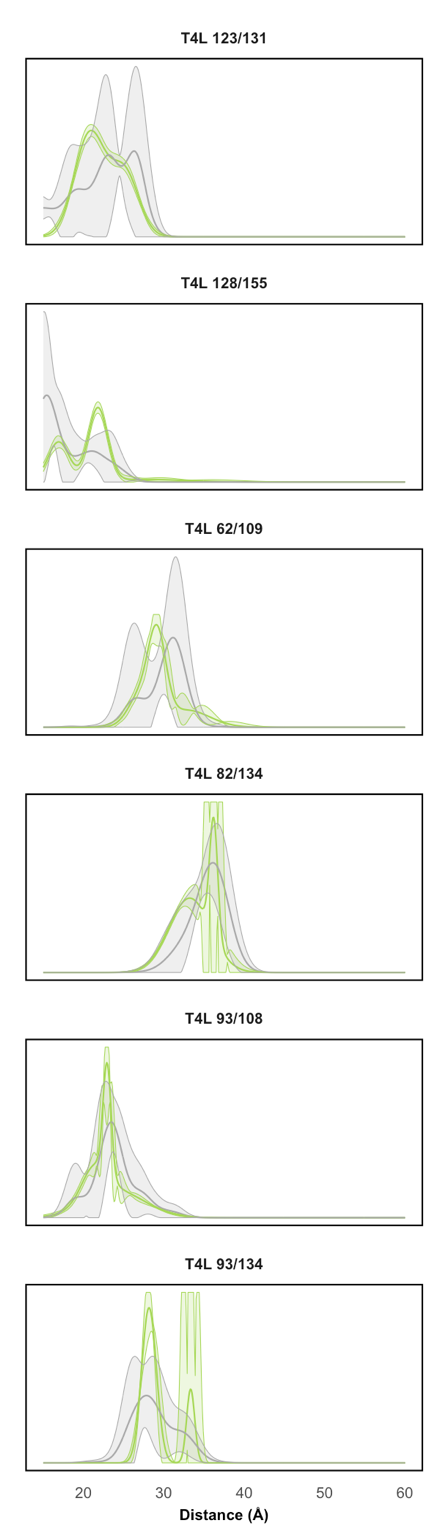

Supplement: S8 Fig — Distributions obtained using GLADDvu and RosettaDEER are shown in green and grey, respectively. Confidence bands for RosettaDEER depict the five best sets of pseudo-rotamers. (TIFF) [file pcbi.1009107.s008.tiff]

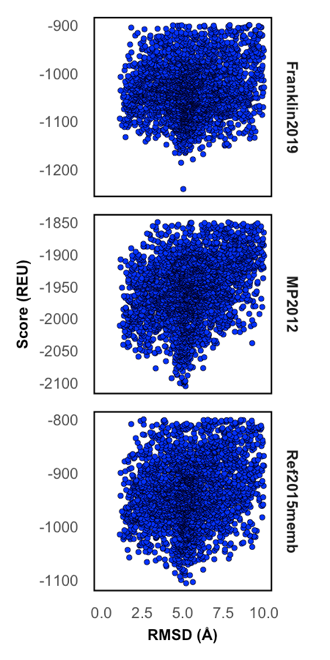

Supplement: S9 Fig — In all three cases, the lowest-energy models are fully occluded from both sides of the membrane. RMSD is measured from the inward-facing crystal structure (PDB: 6FHZ); the first 50 residues were omitted. (TIFF) [file pcbi.1009107.s009.tiff]
