# Supplementary material for: Methodology for rigorous modeling of protein conformational changes by Rosetta using DEER distance restraints
Source: PLoS Comput Biol. 2021 Jun 16;17(6):e1009107. doi: 10.1371/journal.pcbi.1009107 (PMC8238229; doi:10.1371/journal.pcbi.1009107)
Supplement: S1 Table — (DOCX) [file pcbi.1009107.s010.docx]

| N. restraints | Set 1 | Set 2 | Set 3 | Set 4 |
| --- | --- | --- | --- | --- |
| 1 | 120/269 | 120/413 | 240/442 | 215/318 |
| 2 | 215/318 | 215/318 | 186/348 | 186/269 |
| 3 | 120/413 | 134/269 | 215/394 | 240/442 |
| 4 | 215/394 | 215/394 | 196/348 | 120/413 |
| 5 | 134/269 | 186/348 | 215/442 | 240/318 |
| 6 | 215/442 | 215/442 | 120/269 | 134/269 |
| 7 | 186/269 | 186/269 | 215/318 | 186/348 |
| 8 | 240/318 | 240/318 | 186/269 | 196/348 |
| 9 | 186/348 | 196/348 | 240/318 | 215/442 |
| 10 | 240/442 | 240/442 | 120/413 | 120/269 |
