# Supplementary material for: Methodology for rigorous modeling of protein conformational changes by Rosetta using DEER distance restraints
Source: PLoS Comput Biol. 2021 Jun 16;17(6):e1009107. doi: 10.1371/journal.pcbi.1009107 (PMC8238229; doi:10.1371/journal.pcbi.1009107)
Supplement: S5 Appendix — (DOCX) [file pcbi.1009107.s015.docx]

**Appendix 5: Supplemental Methods**

This section discusses the step-by-step process of 1) obtaining pseudo-rotamers by multilateration from a set of DEER data and 2) using those pseudo-rotamers for modeling. We used a development version of Rosetta throughout this work (version 19c0a96ab1533289522c586f36c8ae3b6da052bd).

**5.1. Formatting DEER data for use by Rosetta**

Prior to modeling, the DEER decay data must first be formatted by the user. The RosettaDEER module does not need these data to be background corrected. However, it does require that the data be correctly phased and time corrected; we used GLADDvu for both steps. Once the phased, time-corrected data are saved as a text file, they can be reformatted and collected into one or more files for Rosetta.

The file or files with the DEER data that will be used by Rosetta must be reformatted in a specific way. Each DEER trace must be assigned a unique ID by the user, and information pertaining to each DEER trace is provided using three different types of lines. First, each DEER trace must consist of at least one descriptor line. These lines start with the phrase "DESC" and describe both which residues are spin labeled and which spin label was used. For example, the descriptor line for a DEER trace measured between residues 94 and 123 using MTSSL, and that has been assigned the ID number 1, would be written as follows:

DESC 1 DEFAULT 94 DEFAULT 123

Each line is assigned to a specific DEER trace (identified by the number in the second column) and states which residues are involved in the measurement. The odd-numbered columns (the third and fifth columns) state which spin label depiction is being used, and the even-numbered columns (the fourth and sixth columns) state which residues in the protein structure are spin labeled. In this manuscript, we restrict all DEER measurements to two residues. However, additional residues can be added to this line in the event that the data were collected between three or more residues (for example, in a homotrimer or homotetramer). Additionally, although this manuscript is restricted to spin labeling monomers, detailed descriptions of which residues are spin labeled in homo- and hetero-oligomers can be defined by writing "93B" to, for example, to indicate that the 93rd residue of chain B was spin labeled.

Second, the DEER data itself is passed referring to the unique ID of each DEER trace, which is again in the second column. These lines are formatted as follows:

DECAY 1 -0.024 0.991484

DECAY 1 -0.016 0.995977

DECAY 1 -0.008 0.999551

DECAY 1 0.000 1.0

DECAY 1 0.008 0.998274

DECAY 1 0.016 0.99528

DECAY 1 0.024 0.991384

DECAY 1 0.032 0.98499

DECAY 1 0.040 0.978854

Here each row is a data point collected in the DEER trace, and the third and fourth columns contain the time points (in microseconds) and normalized echo intensities of each point, respectively. There is no limit to the number of time points that can be passed to Rosetta this way.

Finally, additional options can be passed to the protocol specific to any specific DEER trace. These lines, which are not mandatory, describe parameters such as backbone dynamics (which we inferred from the crystallographic B-factors using equations 2 and 3), noise in the imaginary component of the data, and the type of intermolecular background used to fit the DEER signal. These three options can be provided to Rosetta by including the following lines in the file:

INFO 1 NOISE 0.00057976

INFO 1 STDEV 2.21576

INFO 1 BCKG_TYPE NON_3D

These lines would respectively inform Rosetta of 1) the noise anticipated from the imaginary component, 2) the backbone dynamics anticipated from the crystallographic B-factors, and 3) the type of intermolecular background used to model the DEER signal (in this case, a non-three-dimensional background; by default, a three-dimensional background is used).

**5.2. Running the Rosetta multilateration algorithm using RosettaScripts**

The algorithm is run using RosettaScripts. The following XML file is sufficient to execute the multilateration algorithm, and the resulting pseudo-rotamers are printed directly in the terminal (rather than written to a file):

<ROSETTASCRIPTS>

<TASKOPERATIONS>

</TASKOPERATIONS>

<SCOREFXNS>

</SCOREFXNS>

<FILTERS>

</FILTERS>

<MOVERS>

<SwitchResidueTypeSetMover name="cen" set="centroid"/>

<DEEROptimizeCoordsMover name="deer" />

</MOVERS>

<APPLY_TO_POSE>

</APPLY_TO_POSE>

<PROTOCOLS>

<Add mover="cen" />

<Add mover="deer" />

</PROTOCOLS>

</ROSETTASCRIPTS>

The script is then executed from the terminal using the following command:

~/rosetta/main/source/bin/rosetta_scripts.linuxgccrelease -in:file:s <model.pdb>

-parser:protocol <script.xml> -epr_deer:input_files <file1.txt> <file2.txt> <...>

-jd2:no_output -nstruct 1 > output.txt

The algorithm requires a protein structural model (model.pdb), the formatted DEER data (file1.txt and file2.txt), the XML script (script.xml), and a predefined number of outputs (passed using the -nstruct flag). The resulting pseudo-rotamers are then written to output.txt, along with their sum-of-squared residuals and their computed Akaike information criteria-corrected (see Equation 1).

**5.3. Formatting the pseudo-rotamer coordinates for conformational change modeling**

The file output.txt contains the entire contents of the command line output generated by RosettaScripts. The pseudo-rotamer coordinates may be isolated by searching for lines starting with the word "COORD" command, e.g., grep ^COORD output.txt. These lines are formatted as follows:

COORD 60 0.230983 (-3.02, -6.38, 4.59) # (50.647, -19.819, 19.931)

COORD 60 0.0596695 (2.066, -7.341, 1.191) # (46.5518, -16.0789, 17.1695)

COORD 60 0.222950 (-2.384, -5.545, 6.00) # (52.0753, -18.803, 20.0425)

COORD 60 0.007290 (-0.49, -5.153, -2.00) # (44.0504, -17.1789, 20.9175)

COORD 60 0.0419102 (0.307, -7.925, -1.5) # (44.034, -17.946, 18.092)

COORD 60 0.278308 (-5.71, -5.438, -3.02) # (43.6287, -21.7916, 23.5483)

COORD 60 0.0562331 (-1.97, -5.785, -1.45) # (44.6702, -18.7548, 21.0421)

COORD 60 0.102652 (-1.82, -5.937, 5.713) # (51.6649, -18.5373, 19.4806)

COORD 61 0.0784133 (-7.095, -5.243, 2.08) # (44.6746, -15.7433, 35.7542)

Here each line corresponds to a pseudo-rotamer, with the second column being the spin-labeled residue and the third column being its mass or probability. Columns 4-6 are the X, Y, and Z positions of the unpaired electron depicted by the pseudo-rotamer. These coordinates are transformed into the residue's local coordinate frame and will be used by Rosetta for conformational change modeling below. Columns 8-10 are the X, Y, and Z positions of the unpaired electron in the global coordinate frame of the protein and can be used to compute pairwise distance distributions outside of Rosetta.

These coordinates must be slightly modified before being passed to Rosetta. First, the commas and parentheses must be removed. Optionally, columns 7-10 can be discarded at this point. Second, an ID must be assigned to each ensemble of pseudo-rotamers by introducing a new second column as follows:

COORD 1 60 0.230983 -3.02 -6.38 4.59

COORD 1 60 0.0596695 2.066 -7.341 1.191

COORD 1 60 0.222950 -2.384 -5.545 6.003

COORD 1 60 0.007290 -0.491 -5.153 -2.001

COORD 1 60 0.0419102 0.307 -7.925 -1.5

COORD 1 60 0.278308 -5.71 -5.438 -3.02

COORD 1 60 0.0562331 -1.97 -5.785 -1.45

COORD 1 60 0.102652 -1.82 -5.937 5.713

COORD 1 61 0.0784133 -7.095 -5.243 2.08

This column provides an ID to the overall pseudo-rotamer ensemble, thus allowing multiple such ensembles to be used for modeling should the need arise. We did not use this functionality in this manuscript. Finally, as was the case for the file containing the DEER data discussed in section 2.1., a descriptor file must be added:

DESC 1 1.0

This assigns a weight (in column three) to the pseudo-rotamer ensemble with the ID listed in column two.

**5.4. Modeling conformational changes in PfMATE using fragment insertion**

Conformational change modeling in PfMATE was carried out using fragment insertion as implemented in RosettaScripts. Fragments were obtained from the Robetta web server with homologs removed. The resulting XML script is as follows:

<ROSETTASCRIPTS>

<TASKOPERATIONS>

</TASKOPERATIONS>

<SCOREFXNS>

<ScoreFunction name="sfxn" weights="score3" symmetric="0"/>

</SCOREFXNS>

<FILTERS>

</FILTERS>

<MOVERS>

<SingleFragmentMover name="fragmover" fragments="3VVN_03_05.200_v1_3">

<MoveMap>

<Span begin="1" end="50" chi="0" bb="1"/>

<Span begin="51" end="240" chi="0" bb="0"/>

<Span begin="241" end="270" chi="0" bb="1"/>

<Span begin="271" end="461" chi="0" bb="0"/>

</MoveMap>

</SingleFragmentMover>

<GenericMonteCarlo name="mc" mover_name="fragmover" trials="5000"

temperature="1.0" scorefxn_name="sfxn"/>

</MOVERS>

<APPLY_TO_POSE>

</APPLY_TO_POSE>

<PROTOCOLS>

<Add mover="mc"/>

</PROTOCOLS>

</ROSETTASCRIPTS>

We executed this XML script from the command line as follows:

~/rosetta/main/source/bin/rosetta_scripts.linuxgccrelease -in:file:s 3vvn.pdb

-parser:protocol <script.xml> -nstruct 1

The resulting models may then be scored using DEER data as follows:

~/rosetta/main/source/bin/score_jd2.linuxgccrelease -in:file:s <model.pdb>

-epr_deer:input_files <file1.txt> <file2.txt> <...> -epr_deer:coords_files <file.txt>

-score:set_weights epr_deer_score 1 -score:weights none

The pseudo-rotamers obtained using multilateration were provided to this protocol using the epr_deer:coords_files flag. Additionally, the DESC lines of the files containing the DEER data were modified so that instead of using DEFAULT pseudo-rotamers, they used CUSTOM pseudorotamers:

DESC 1 CUSTOM 94 CUSTOM 123

When default pseudo-rotamers were used, the epr_deer:coords_files flag was not passed to Rosetta.
